# Integrating data to assess occupancy patterns of an endangered bumble bee

**DOI:** 10.1111/cobi.14458

**Published:** 2025-02-25

**Authors:** Kristen S. Ellis, Clint R. V. Otto, Larissa L. Bailey, Tamara A. Smith, Steven Choy, Lauren Hatch

**Affiliations:** ^1^ Northern Prairie Wildlife Research Center U.S. Geological Survey Jamestown North Dakota USA; ^2^ Department of Fish, Wildlife and Conservation Biology Colorado State University Fort Collins Colorado USA; ^3^ Graduate Degree Program in Ecology Colorado State University Fort Collins Colorado USA; ^4^ Minnesota‐Wisconsin Ecological Services Field Office U.S. Fish and Wildlife Service Bloomington Minnesota USA

**Keywords:** autologistic model, bee conservation, dynamic occupancy, extirpation risk, metapopulation dynamics, pollinator decline, conservación de abejas, declinación de polinizadores, dinámica de ocupación, dinámicas metapoblacionales, modelo autologístico, riesgo de extirpación

## Abstract

There is growing interest in integrating community science data with structured monitoring data to estimate changes in distribution patterns of imperiled species, including pollinators. However, significant challenges remain in determining how unstructured community science data should be incorporated into formal analyses of species distributions. We developed a dynamic framework for combining community science and structured monitoring data of bumble bees to estimate changes in occupancy of rusty‐patched bumble bees (*Bombus affinis*), a federally endangered species in the United States. We applied traditional metapopulation theory and accounted for imperfect detection to estimate site‐specific extirpation risk and colonization rates across the known distribution of *B. affinis* in the Upper Midwest (USA). Despite a 144% increase in presence‐only detections from 2017 to 2022, occupancy probabilities and the estimated number of occupied sites remained static or declined slightly across a 4‐state region during this period. Our results provide preliminary evidence that the probability of local extirpation risk of *B. affinis* increased in response to drought, but that effect was tempered with a high number of neighboring patches occupied by *B. affinis* (i.e., rescue effect). Our framework can be used by managers to track population recovery goals for *B. affinis* and other bumble bees of conservation concern. In addition, our study highlights the importance of accounting for imperfect detection and addressing spatial sampling biases in bumble bee monitoring efforts, particularly those for which a portion of the monitoring data are generated from community science projects.

## Abstract (es)

Integración de los datos para analizar los patrones de ocupación de un abejorro en peligro de extinción

**Resumen:**

Cada vez hay un mayor interés por integrar los datos de la ciencia comunitaria con los datos de monitoreo estructurado para estimar los cambios en los patrones de distribución de las especies en peligro, incluidos los polinizadores. Sin embargo, todavía hay problemas importantes a la hora de determinar cómo deben incorporarse los datos no estructurados de la ciencia comunitaria a los análisis formales de la distribución de las especies. Desarrollamos un marco dinámico para combinar datos de ciencia comunitaria y de monitoreo estructurado de abejorros con el fin de estimar los cambios en la ocupación de *Bombus affinis*, una especie en peligro de extinción en Estados Unidos. Aplicamos la teoría metapoblacional tradicional y tuvimos en cuenta la detección imperfecta para estimar el riesgo de extirpación y las tasas de colonización específicas de cada lugar en la distribución conocida de *B. affinis* en el Medio Oeste Superior (EE. UU.). A pesar de un aumento del 144% de 2017 a 2022 en las detecciones de presencia, las probabilidades de ocupación y el número estimado de sitios ocupados se mantuvieron estáticos o disminuyeron ligeramente en una región de cuatro estados durante este período. Nuestros resultados proporcionan evidencia preliminar de que la probabilidad de riesgo de extirpación local de *B. affinis* aumentó en respuesta a la sequía, pero ese efecto se atenuó con un alto número de parches vecinos ocupados por *B. affinis* (es decir, efecto de rescate). Los gestores pueden utilizar nuestro marco para realizar un seguimiento de los objetivos de recuperación de la población de *B. affinis* y otros abejorros de interés para la conservación. Además, nuestro estudio subraya la importancia de tener en cuenta la detección imperfecta y abordar los sesgos de muestreo espacial en los esfuerzos de seguimiento de abejorros, en particular aquellos para los que una parte de los datos de seguimiento se generan a partir de proyectos científicos comunitarios.

## INTRODUCTION

Online community science platforms, such as iNaturalist and eBird, produce vast amounts of species occurrence records that are available for public use (Bonney et al., 2014; Brown & Williams, 2019; Pocock et al., 2018; Sullivan et al., 2009). Community science efforts can range from structured surveys that contain details on sampling effort (e.g., Breeding Bird Survey [Sauer & Link, 2011]) to unstructured observations of species at a specific time and location (e.g., iNaturalist). The temporal and spatial coverage of species occurrence records collected by community members (inclusive of any volunteer participant) (Cooper et al., 2021) can provide researchers with a wealth of data that are often unmatched by directed monitoring efforts. Additionally, community science programs enhance community empowerment, offer colearning opportunities between community members and scientists, and improve human connections with nature (Adler et al., 2020; Balazs & Morello‐Frosch, 2013). However, the reliability of community science data in informing patterns of biodiversity and ecological relationships depends on an understanding of spatial or taxonomic biases and variation in sampling efforts (Brown & Williams, 2019; Geurts et al., 2023).

Nonrandom or inconsistent sampling efforts are typical in community science because there is often a lack of sampling guidelines, resulting in concentrations of data in areas that are easy to access or near urban centers and variability in the scope, duration, and intensity of surveys (Geurts et al., 2023). Therefore, methods to account for spatial biases or observer variability in detection–nondetection modeling are commonly used (Van Strien et al., 2013). When biases are appropriately accounted for, integrating community science data with expert surveys can improve the accuracy of species distribution models and fill important spatial or temporal gaps when making inferences on distributional dynamics (Guzman et al., 2021; Robinson et al., 2020; Van Strien et al., 2013). This additional data can be particularly informative for rare or declining species when assessing their distributions across broad spatial regions (e.g., Ellis et al., 2023; Lin et al., 2022; Whitenack et al., 2023). For example, community science data can enhance the understanding of detection processes of rare species by providing information on when and where observers have detected closely related species if the rare species was not detected.

Bumble bees (*Bombus* spp.) have become the focus of several community science programs due to widespread population declines across North America and Europe (Cameron et al., 2011; Goulson et al., 2008; Grixti et al., 2009). Programs including Bumble Bee Atlas (www.bumblebeeatlas.org) and Bumble Bee Watch (www.bumblebeewatch.org), among others, were developed to collect bumble bee monitoring data in response to these population declines. There are multiple interacting stressors linked to bumble bee declines, including habitat loss, pathogen transmission, and pesticides (Cameron et al., 2011; Goulson et al., 2015; Kerr et al., 2015; Szabo et al., 2012). Climate change and drought can exacerbate these stressors, presumably by limiting the quantity and quality of floral resources and creating phenological mismatches between bees and flower emergence (Graves et al., 2020; Janousek et al., 2023; Soroye et al., 2020). Both climate and land use are important determinates of European bumble bee ranges, and climate plays a large role in shaping the current distribution of declining bumble bees (Marshall et al., 2018; Whitehorn et al., 2022). Changing global climatic factors such as air and water temperature and increasing drought risk (Reidmiller et al., 2017) will likely accelerate the rate of bumble bee declines and extinctions (Cameron & Sadd, 2020; Jackson et al., 2022). Variations in the intensity of interacting stressors across space and species‐specific responses to certain stressors (Szabo et al., 2012) highlight the need for effective bumble bee monitoring. In the United States, a national program to monitor native bees is lacking, despite critical contributions of bees to crop and native plant pollination (Woodard et al., 2020). Further, bumble bees are relatively large insects, and certain species have distinct features; therefore, nonlethal survey methods that community scientists are able to implement can be robust and effective (Falk et al., 2019; Portman et al., 2020; Satyshur et al., 2023; Suzuki‐Ohno et al., 2017). This provides an opportunity for community science data to play a role in widespread monitoring, given limitations of locally focused efforts to monitor bumble bees (Satyshur et al., 2023).

The rusty‐patched bumble bee (*Bombus affinis*) (RPBB) was listed as a federally endangered species in the United States (USFWS, 2017) and Canada (ECCC, 2012) following precipitous population declines. Historically, RPBBs were numerous and widespread across the Midwest and eastern United States. Recent occurrence records indicate that the species occurs in approximately 5% of locations in its former range (USFWS, 2016) and is broadly concentrated near urban centers in the Upper Midwest (USFWS, 2021). Further, its fine‐scale distribution does appear to be associated with human‐developed lands (Boone et al., 2023b). It is critical to determine whether distribution patterns reflect nonrandom survey efforts in locations that are easily accessible by community scientists or whether natural spaces near urban centers may serve as refugia across their range for this endangered species. The U.S. Fish and Wildlife Service (USFWS) Recovery Plan for Rusty‐Patched Bumble Bee and the recovery implementation strategy list specific occupancy targets for downlisting or delisting the species and specific science needs for successful recovery (USFWS, 2021, 2022a). Two of the major science needs include a framework for estimating changes in occupancy that accounts for spatial sampling bias and false absences and investigations into RPBB habitat requirements and response to geographic‐specific stressors (USFWS, 2021).

We combined data from common bumble bee species collected from community science efforts and a presence‐only data set of RPBBs, managed by the USFWS, to investigate occupancy dynamics of RPBBs from 2017 to 2022. Our objective was to develop a model managers could use to produce annual estimates of RPBB occupancy to track recovery. Because conservation practitioners are also interested in understanding factors influencing the underlying occupancy dynamics, our model estimates annual extirpation and colonization probabilities and relates these parameters to drought and the number of occupied neighboring grid cells. We sought to develop a method for estimating changes in RPBBs through time and flexibility for incorporating other forms of data to determine how environmental stressors affect occupancy dynamics in the absence of structured monitoring data.

## METHODS

### Occupancy as a state variable

**FIGURE 1:**
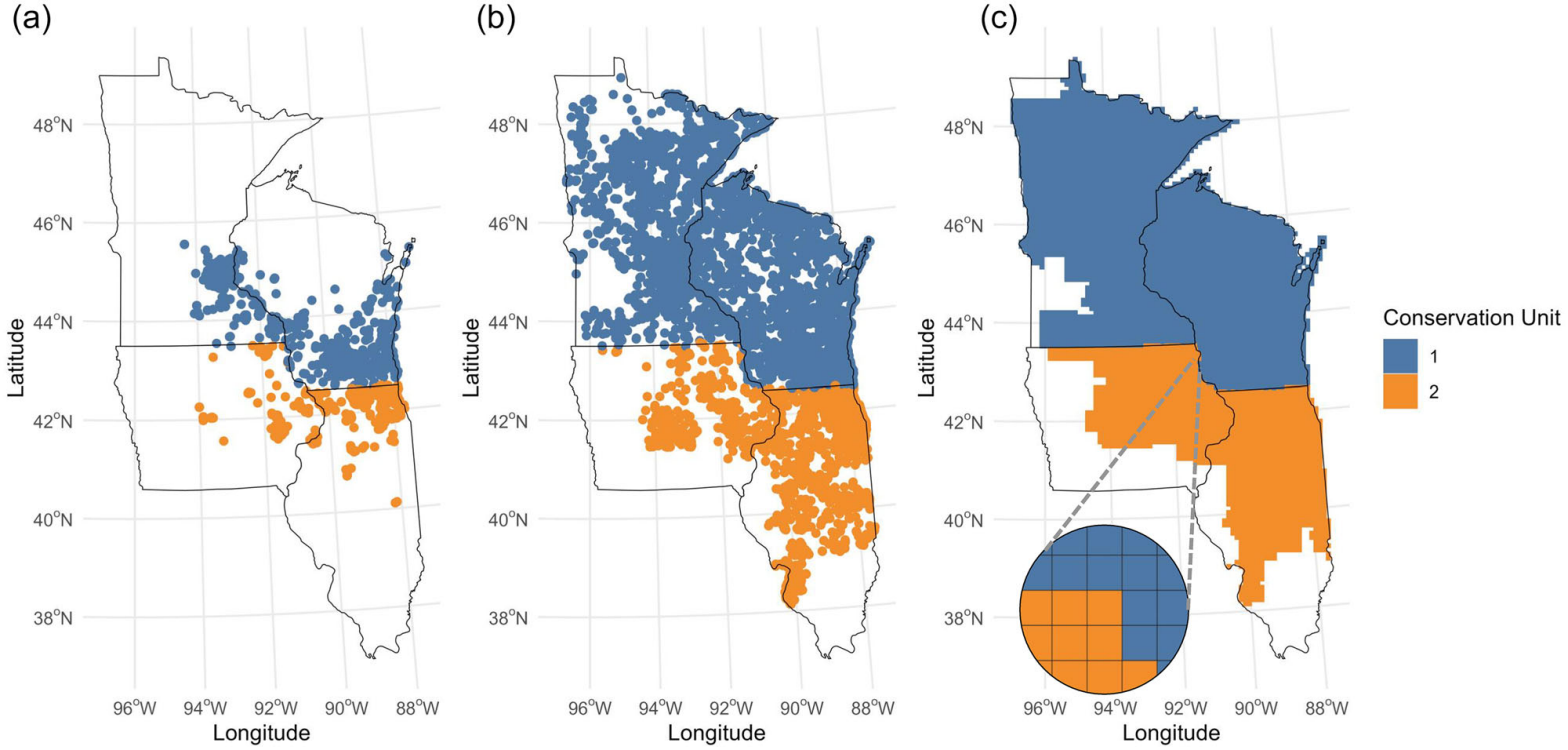
In Minnesota, Wisconsin, Iowa, and Illinois, (a) location of detection points of rusty‐patched bumble bee (*Bombus affinis*) (*n* = 7324) from 2017 to 2022 and (b) location of detection points of common bumble bees (*Bombus* spp.) (*n* = 54,179) identified by community scientists from 2017 to 2022 in 10 × 10‐km grid cells in each conservation unit (c).

The USFWS is leading recovery efforts and has a specific interest in tracking occupancy trends of RPBBs across 5 conservation units that span the historic distribution of the species (USFWS, 2021). The Recovery Plan for Rusty‐Patched Bumble Bee includes specific conditions related to occupancy for downlisting and delisting the species. First, occupancy must be stable or increasing across all conservation units over a minimum of 5–10 years (USFWS, 2021). Second, the USFWS lists occupancy targets for each of the conservation units and uses a 10 × 10‐km grid across the historic range to track RPBB detections and presumed occupancy (available from https://www.arcgis.com/home/webmap/viewer.html?webmap=feab9e66608840818795c2dfef93993b&extent). The 10 × 10‐km grid cells represent the unit of inference for our occupancy models (Figure 1). We assumed that observers surveyed potential *Bombus* habitats (i.e., herbaceous grasslands, open areas, forests, and developed lands with floral resources) in a 10 × 10‐km grid cell during a given week. Because the amount and locations of surveyed habitats in each cell were not recorded, we assumed observers surveyed a representative sample of potential floral resources each week when a detection occurred. Our occupancy estimates therefore reflect the probability of RPBB occupancy in *Bombus* habitats in a given grid cell.

**FIGURE 2:**
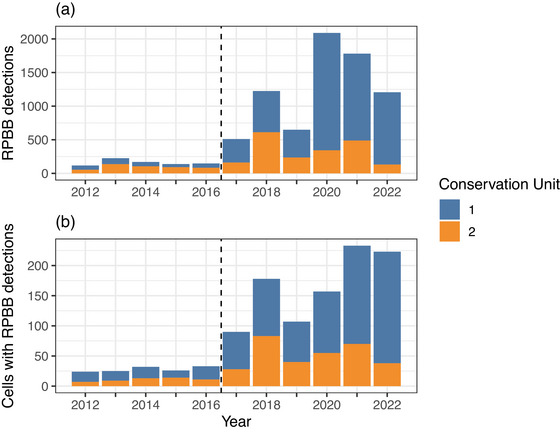
Rusty‐patched bumble bee (*Bombus affinis*) (RPBB) (a) detections and (b) unique grid cells with detections from 2012 to 2022 in the Upper Midwest, United States (dashed vertical line, year the rusty‐patched bumble bee was listed as endangered in the United States). Our analysis includes data from 2017 to 2022.

The USFWS manages a data set of RPBB detections, consisting of 20,667 records from 1900 to 2022 (Figures 1a & 2). The USFWS database was created by extracting the RPBB records from the Bumble Bees of North America database, supplemented by a combination of observations collected for a variety of purposes. The database includes RPBB observations submitted to Biodiversity Information Serving Our Nation (BISON) and those from entities holding scientific research permits under section 10(a)1(A) of the Endangered Species Act (ESA), state natural resource agencies (e.g., Wisconsin Bumble Bee Brigade), and Bumble Bee Atlas projects. The database also includes verified or science‐grade observations submitted to BumbleBeeWatch.org, iNaturalist.org, and BeeSpotter.org and those directly reported to USFWS. The USFWS receives unobscured RPBB records from these sources to maintain location accuracy. This collection of detection records is being used to track changes in RPBB occupancy and evaluate progress toward recovery goals. This presence‐only data set lacks records for which observers searched for bumble bees but did not detect RPBBs, which makes inferring absence challenging. Occurrence records are displayed on the USFWS Priority Survey Grid Map (refer to the website above) and note which grid cells have known RPBB occurrences.

### Data augmentation

We addressed the lack of nondetection data of RPBBs by combining detection data of 23 *Bombus* species gathered from multiple community science platforms (Figure 1b). We initially obtained 53,505 detection records from the USFWS Bee Tool (www.TheBeeTool.com), which contains spatially explicit observations of native bees from a variety of data sources. Our extraction of *Bombus* records from the USFWS Bee Tool included data from iNaturalist, BISON, the Field Museum of Natural History, the Illinois Natural History Survey, and Bumble Bee Watch. We selected only science‐grade observations for all iNaturalist records. We also obtained 12,722 *Bombus* records from the Bumble Bee Brigade (https://wiatri.net/inventory/bbb/), a Wisconsin‐based community science monitoring program coordinated by the Wisconsin Department of Natural Resources. We considered obtaining data from the Bumble Bee Atlas, but data collection efforts in the Midwest were only initiated after 2020. We did not obtain RPBB detection records from sources that were already archived in the USFWS database. We eliminated records without latitude, longitude, or sampling date information, and we assumed that observers reported all *Bombus* species detected. We overlayed the RPBB (i.e., USFWS data set) and other *Bombus* records (i.e., Bee Tool and Bumble Bee Brigade records) with the USFWS 10×10 km Priority Grid Cell Map, focusing on states in RPBB Conservation Units 1 and 2 (i.e., Minnesota, Wisconsin, Illinois, and Iowa) (Figure 1c). We constructed RPBB detection histories by addressing whether RPBB or a common bumble bee was detected in a particular grid cell during a given week (see below).

### Statistical analyses

To estimate occupancy and its dynamic components (extirpation and colonization probabilities) while adjusting for imperfect detection, we used a hierarchical parameterization of a dynamic occupancy model (MacKenzie et al., 2017; Royle & Kéry, 2007). We applied dynamic occupancy modeling to opportunistic bumble bee data because this framework can be used to mitigate some biases associated with unequal sampling effort (Van Strien et al., 2013) while estimating the dynamic properties of occupancy that govern changes in species distributions. We used bumble bee occurrence data from 2017 to 2022. Each annual season contained aggregated records from 1 June to 30 September grouped into weekly surveys to allow for the estimation of detection probabilities. We aggregated occurrence records into weekly intervals to limit detection probabilities from being dominated by a few well‐sampled grids. We assumed that if ≥1 common bumble bees were observed, RPBB detections would also have been recorded by community scientists. Therefore, common bumble bee observations in the absence of RPBB detections were considered nondetections of RPBB. For each grid cell *i* and week *j*, we determined if ≥1 RPBB was detected (1 = RPBB detected), ≥1 common bumble bee was detected but RPBB was not (0 = RPBB not detected), or neither RPBBs nor common bumble bees were detected (NA = missing data).

We used a dynamic occupancy model to estimate the probability of initial grid cell occupancy ($\psi_{1}$); the probability of grid cell colonization in year *t* + 1 ($\gamma_{t}$), given it was unoccupied in year *t*; the probability of grid cell extirpation in year *t* + 1 ($\varepsilon_{t}$), given it was occupied in year *t*; and the probability of detection in week *j*, given the grid cell was occupied ($p_{j,t}$). The latent occupancy state of grid *i* was described by $z_{i,1}\;∼\;\mathrm{Bernoulli}\left(\psi_{i,1}\right)$ in year one, 2017. In subsequent years *t* = 2, 3,…, *T*, $z_{i,t}$ was a function of dynamic parameters

(1) $$z_{i,t}∼\mathrm{Bernoulli}\left[z_{i,t-1}\left(1-\varepsilon_{i,t-1}\right)+\gamma_{i,t-1}\left(1-z_{i,t-1}\right)\right],$$

where $\varepsilon_{t}$ can alternatively be written as its complement (i.e., persistence probability) (Royle & Kéry, 2007). We derived the finite sample occupancy (Royle & Kéry, 2007) as

(2) $$\psi_{t}^{\mathrm{fs}}=\frac{1}{R}\;\sum_{i}z_{i,t}$$

with the subset of surveyed (R) grids in each year. Estimates of finite sample occupancy allowed us to make inferences on species occurrence at sampled grid cells and to compare them with inferences from a larger study area from which a selection of cells had been sampled (unconditional occupancy probability) (Royle & Kéry, 2007). In our case, the finite sample occupancy corresponded to only grid cells where data on detection or nondetection or both were available for a given year, whereas the unconditional occupancy probability applied to all grid cells, sampled and unsampled, across Minnesota, Wisconsin, Illinois, and Iowa (Figure 1c).

Although inferring land‐cover associations with RPBB occupancy was not a focal point of our analyses, we modeled initial occupancy probabilities for 2017 as a function of land‐cover covariates, which we obtained from the 2019 National Land Cover Database (Dewitz & U.S. Geological Survey, 2021). We calculated the proportion of certain land‐cover types in each 10 × 10‐km grid cell, including cultivated crops, grassland or herbaceous, pasture or hay, shrub or scrub, and development. In a preliminary analysis, we used a simplified dynamic occupancy model with year‐specific intercepts for colonization and extirpation probabilities and a time‐varying structure for detection probability (detection probability varied by survey week with year‐specific intercepts) to identify the most supported land‐cover variables explaining initial occupancy. Subclassifications of development (open space, low intensity, medium intensity, and high intensity) were highly correlated (|*r*| > 0.9), so we considered combinations of development subclassifications (e.g., sum of open space, low, and medium intensity). Other land‐cover types were not highly correlated (|*r*| < 0.6). We evaluated relative support among candidate models with widely applicable information criterion (WAIC) (Watanabe, 2010) and identified 3 land‐cover predictors that best explained initial occupancy including grassland or herbaceous (herb), pasture or hay (hay), and the sum of developed open space, low intensity, and medium intensity (DEV) (Appendix S1). We allowed initial occupancy to vary between conservation units (CU) by using an indicator variable (Conservation Unit 1 = 1 and Conservation Unit 2 = 0). Initial occupancy probabilities ($\psi_{i,1}$) in the full model were estimated as

(3) $$\mathrm{logit}\;\left(\psi_{i,1}\right)=\alpha_{\psi }+\beta_{\psi 1}\mathrm{her}b_{i}+\beta_{\psi 2}\mathrm{DE}V_{i}+\beta_{\psi 3}\mathrm{ha}y_{i}+\beta_{\psi 4}CU_{i}$$

at grid cell *i* with $\alpha_{\psi }$ as the intercept.

We predicted that extirpation and colonization probabilities would vary as a function of the occupancy status of neighboring grids at time *t* − 1 (i.e., potential sources of colonists) (Hanski, 1998). We modeled spatial neighborhood effects using autologistic functions that incorporated the estimated occupancy status of neighboring grids (Eaton et al., 2014; Yackulic et al., 2012). Specifically, we estimated the number of occupied grids (${\hat{\theta }}_{i,t}$) in the neighborhood of grid cell *i* in year *t* as

(4) $${\hat{\theta }}_{i,t}=\sum_{n\in \left\{N_{i}\right\}}{\hat{z}}_{n,t},$$

where $N_{i}$ is the set of adjacent grid cells (8 total), and ${\hat{z}}_{n,t}$ is the estimated occupancy status of neighboring cell *n* at time *t*. We additionally predicted that extirpation and colonization probabilities would vary as a function of seasonal weather conditions, specifically drought, which has been identified as a stressor for bumble bees (Janousek et al., 2023). We used monthly measures of the Palmer Drought Severity Index (PDSI) from 2017 to 2022 (Abatzoglou et al., 2018) to measure annual drought conditions. Negative values of PDSI indicate dryer conditions, and positive values indicate wetter conditions. We calculated a seasonal PDSI value for each grid cell by averaging monthly values from May to July, when resource availability is directly linked to colony development later in the summer (Malfi et al., 2019). Summarized PDSI values indicated that conditions were, on average, wetter across our study period except for 2021 when the median PDSI was negative (i.e., greater frequency of drought conditions across all surveyed cells) (Appendix S2). Extirpation ($\varepsilon_{i,t}$) and colonization ($\gamma_{i,t}$) probabilities were modeled as

$$\log\mathrm{it}\;\left(\varepsilon_{i,t}\right)=\alpha_{\varepsilon t}+\beta_{\varepsilon 1}{\hat{\theta }}_{i,t-1}+\beta_{\varepsilon 2}\mathrm{PDS}I_{i,t}+\beta_{\varepsilon 3}CU_{i}$$

and

(5) $$\log\mathrm{it}\;\left(\gamma_{i,t}\right)=\alpha_{\gamma t}+\beta_{\gamma 1}{\hat{\theta }}_{i,t-1}+\beta_{\gamma 2}\mathrm{PDS}I_{i,t}+\beta_{\gamma 3}CU_{i}$$

with the estimated number of occupied neighboring cells from the previous year (${\hat{\theta }}_{i,t-1}$) and year‐specific intercepts, $\alpha_{\varepsilon t}$ and $\alpha_{\gamma t}$. We also included the effects of conservation units on extirpation and colonization probabilities similar to initial occupancy.

We expected that detection probabilities would vary within the season, primarily due to changes in *Bombus* colony sizes (Otto et al., 2023; Williams et al., 2014). Our estimates of detection probability should be interpreted as the probability of detecting RPBBs during searches of potential bumble bee habitats conducted during a week, given RPBBs occurred in the 10 × 10‐km cell during the season. We modeled survey week (18 weeks starting 1 June and ending 30 September) as an autoregressive random effect to describe temporal patterns of detection probability. We included the seasonal count of *Bombus* detections (not including RPBBs) from community science data in each grid cell to account for the sampling effort. We expected lower RPBB detection probabilities when detections of other *Bombus* species were low. Detection probabilities ($p_{i,j,t}$) at grid cell *i* during week *j* in year *t* were modeled as

(6) $$\begin{array}{ccc} \log\mathrm{it}\;\left(p_{i,j,t}\right) & = & \alpha_{p,j,t}+\beta_{p}\mathrm{coun}t_{i,t},\\ & & \alpha_{p,j,t}\;∼\;\mathrm{normal}\left(\alpha_{p,j-1,t},\sigma_{p}\right) \end{array}$$

with a random intercept for survey week and year ($\alpha_{p,j,t}$) that linearly depended on the estimate from the previous week and $\sigma_{p}$ as a variance term allowing for weekly stochastic deviations.

We fit models in a Bayesian framework with JAGS (Plummer & others, 2003) in R 4.2.2 (R Core Team, 2021) via the jagsUI package (Kellner, 2019). We used conventional vague priors for all intercepts (α) and covariate effects (β) using normal distributions with a mean of 0 and a standard deviation of 2 (Northrup & Gerber, 2018). We present mean and 95% credible intervals (CIs) for β and used the proportion of posterior distributions that were greater or less than zero for inference on covariate effects, which can be interpreted as the probability that the effect is greater or less than zero (Hobbs & Hooten, 2015). Covariates for initial occupancy, extirpation, colonization, and detection probabilities were standardized to have a mean equal to zero and a variance equal to one, except for $\hat{\theta }$, which was estimated during each iteration. We used 4 chains of 20,000 iterations, including 5000 iterations discarded for burn‐in, each thinned to 5000 samples. We assessed model convergence with a visual examination of trace plots to ensure mixing of chains and the Gelman–Rubin statistic (<1.1) (Gelman & Rubin, 1992).

We evaluated model fit with Bayesian *p* values with the deviance of observed data from $\psi_{i,t}$ and $p_{i,j,t}$ probabilities associated with each iteration compared with the deviance for detections predicted from the model (Broms et al., 2016). Bayesian *p* values near 0.5 indicate an adequate fit, whereas values near 0 or 1 indicate poor model fit (Broms et al., 2016; Kery & Royle, 2020). The Bayesian *p* value for the model did not indicate a lack of fit (*p* = 0.43).

## RESULTS

Our combined data set consisted of 7324 RPBB detection records and 54,179 nondetection records (where common bumble bees were detected but RPBBs were not). There were an average of 1221 RPBB annual records (SD = 602, annual range: 509–2061). Surveyed grid cells averaged 1.3 and 9.5 RPBB and common bumble bee detections in a given year (SD = 7.8 and 32.2), respectively. The average number of RPBB detections per surveyed grid cell per year remained relatively consistent during our study period (range: 0.70–1.8), whereas common bumble bee detections increased (4.0 in 2017 and 12.24 in 2022). After the decision to list RPBB as an endangered species in 2017, the total number of RPBB detections increased. The most detections were in 2020 (Figure 2a; Appendix S3). Similarly, there was a 144% increase in the number of grid cells with RPBB detections from 2017 to 2022. The highest number of known occupied grid cells was recorded in 2021 (Figure 2b; Appendix S3).

**FIGURE 3:**
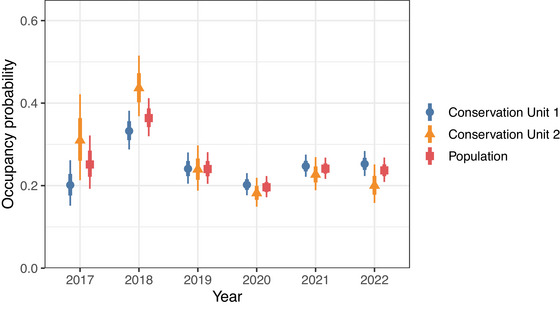
Posterior distributions of conservation unit and population‐level occupancy probabilities for rusty‐patched bumble bee (*Bombus affinis*) from 2017 to 2022 in the Upper Midwest, United States (points, posterior means; thick vertical bars, 66% credible intervals; thin vertical bars, 95% credible intervals).

**FIGURE 4:**
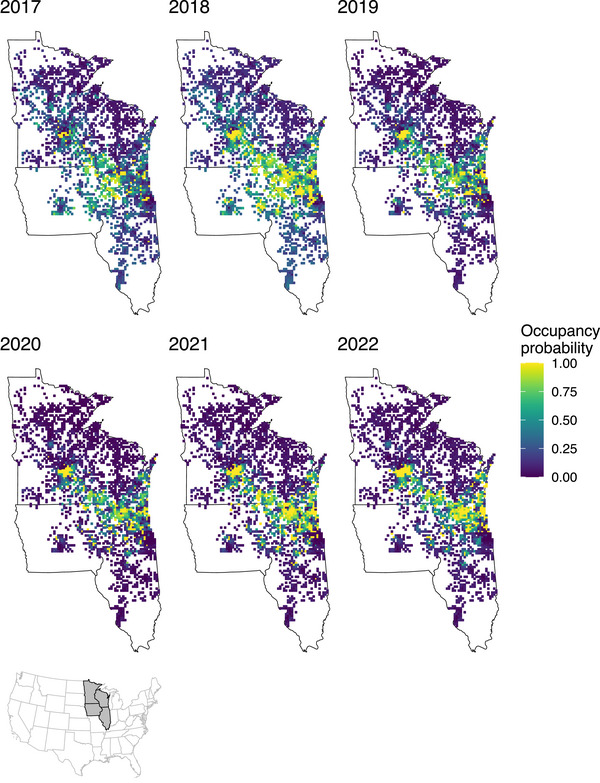
Annual predictions of latent occupancy probability for rusty‐patched bumble bee (*Bombus affinis*) across all surveyed areas in the Upper Midwest, United States from 2017 to 2022. Each pixel represents a 10 × 10‐km grid cell.

Despite the increase in the number of raw detections of RPBBs and number of known occupied grid cells from 2017 to 2022 (Figure 2), estimates of RPBB occupancy were relatively constant, particularly from 2019 to 2022, after a decrease from 2018 to 2019 (Figure 3). Finite‐sample occupancy probabilities ($\psi_{t}^{\mathrm{fs}}$) (conditional on the sampled grid cells) were higher on average than unconditional estimates ($\psi_{t}$), although 95% CIs overlapped, suggesting spatial sampling biases may be present in areas where RPBBs are being detected (${\hat{\psi }}^{\mathrm{fs}}$ across all years = 0.32, 0.23–0.47; $\hat{\psi }$ = 0.26, 0.18–0.39). Annual patterns were consistent between the unconditional (Figure 3) and finite occupancy estimates (Appendix S4) where occupancy was highest in 2017 and 2018, declined slightly in 2019, and remained static thereafter. The estimated average number of occupied grid cells was 609 (annual range: 465–865) (Appendix S5). Even after a considerable increase in the number of RPBB detections in 2020–2022 (Figure 2), the realized distribution of RPBBs was largely confined to southern and eastern Wisconsin, northern Illinois, and southeastern Minnesota (Figure 4). Probabilities of initial occupancy in 2017 varied with land cover and were positively related to proportions of herb ($\beta_{\psi 1}$ = 0.12, 88% of $\beta_{\psi 1}$ was >0), DEV ($\beta_{\psi 2}$ = 0.24, 99% of $\beta_{\psi 2}$ was >0), and hay ($\beta_{\psi 3}$ = 0.85, 100% of $\beta_{\psi 3}$ was >0) (Appendix S6).

**FIGURE 5:**
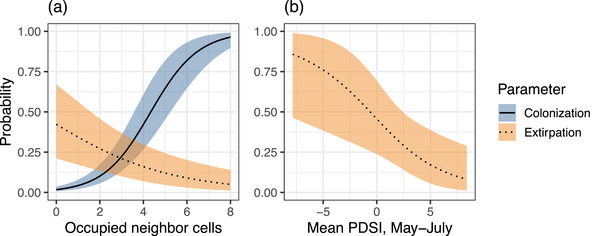
Relationships between probabilities of extirpation and colonization of rusty‐patched bumble bees and the estimated number of occupied neighbor grids and summer drought conditions in the Upper Midwest, United States, for effects with 95% credible intervals that did not overlap zero: (a) response curves calculated for the number of occupied neighbor grids under average drought conditions (Palmer Drought Severity Index [PDSI] = 2.17) and (b) response curves calculated for drought conditions with the average number of occupied neighbor grids ($\hat{\theta }$ = 1.42) All response curves are averaged across years and conservation units.

Most surveyed cells had few occupied neighbors (mean $\hat{\theta }$ across all cells and across cells with at least one RPBB detection in our study period = 1.42 and 3.37, respectively) (Appendix S7). Colonization and extirpation probabilities were related to the number of occupied neighbor grids (Figure 5a) where probabilities of colonization increased ($\beta_{\gamma 1}$ = 0.94, 100% of $\beta_{\gamma 1}$ was >0) and probabilities of extirpation decreased ($\beta_{\varepsilon 1}$ = −0.34, 100% of $\beta_{\varepsilon 1}$ was <0) when more neighbor cells were occupied (Appendix S6; Figure 5a). Extirpation probabilities decreased with PDSI (wetter than average conditions; $\beta_{\varepsilon 2}$ = −0.94, 99% of $\beta_{\varepsilon 2}$ was <0) (Appendix S6; Figure 5b), suggesting that local extirpation risk increased in grid cells with more sustained drought. We did not detect an effect of PDSI on colonization probabilities ($\beta_{\gamma 2}$ was centered around zero) (Appendix S6). Average extirpation probabilities across all years were greater than colonization probabilities at cells with an average number of occupied neighbors (ε = 0.42, 0.04–0.91; γ = 0.09, 0.01–0.25 when $\hat{\theta }$ = 1.42). Colonization and extirpation probabilities varied annually, with the highest probabilities of extirpation and the lowest probabilities of colonization occurring from 2018 to 2019 (Appendices S6 & S8). Colonization probabilities were similar between conservation units; however, extirpation probabilities were lower in Conservation Unit 1 ($\beta_{\varepsilon 3}$ = −0.80, 99% of $\beta_{\varepsilon 3}$ was <0) (Appendices S6 & S8).

Detection probabilities for RPBBs in all years showed midseason peaks (corresponding with mid‐July through late August). Probabilities of detection were low (<0.10) in early and late summer and were relatively similar among years (Appendix S9). The lowest probabilities of detection were in 2019 (mean across all weeks = 0.22) and highest in 2017 (mean across all weeks = 0.27). Unsurprisingly, RPBB detection probabilities were positively related to counts of other *Bombus* detections ($\beta_{p}$ = 0.08, 100% of $\beta_{p}$ was >0) (Appendix S6), suggesting that RPBBs were more likely to be detected in cells where community scientists spent more effort looking for bumble bees.

## DISCUSSION

Our results highlight the utility of combining multiple forms of community science and monitoring data to estimate species occupancy trends of imperiled pollinators. Given simultaneous growth in community science projects for pollinators (e.g., Falk et al., 2019; Satyshur et al., 2023; Suzuki‐Ohno et al., 2017) and the national movement for more structured native bee monitoring in the United States (Woodard et al., 2020), we showed how disparate data sources can be used to inform species recovery efforts of threatened and endangered bumble bees. Although our analyses focused solely on the endangered RPBB, our framework can be applied to other pollinators that have been petitioned for listing under the ESA, such as the American bumble bee (*Bombus pensylvanicus*), southern plains (*Bombus fraternus*), and western bumble bee (*Bombus occidentalis*). In addition to detecting population trends, decision makers ultimately want data from monitoring efforts to elucidate the mechanisms by which species distribution patterns change or species decline (e.g., Grant et al., 2016). Our results provide insight into factors that influence changes in the extirpation and colonization of RPBBs, thereby uncovering factors that shape the distribution of this endangered species.

By incorporating nondetection data, our framework has the benefit of estimating occupancy across a large landscape where sampling effort may be highly variable, and the actual distribution of the species may be obscured by nonrandom and unequal sampling effort. Validating nondetection assumptions and modeling decisions can be useful for identifying biased estimates of species declines that may mislead conservation applications (Guzman et al., 2021). We modeled variation in detection probabilities inherent in community science data by incorporating counts of common *Bombus* species. However, factors influencing the detection and occupancy of *Bombus* species may differ and biases may still persist in our analyses due to varied occurrences and observational processes. For example, relationships vary among detection probabilities and day of year among *Bombus* species (Boone et al., 2023a), likely due to differences in colony phenology. Spatial and temporal variability in common species detections could skew RPBB occupancy and detection estimates if peak worker abundances do not align with RPBB peaks, particularly if surveys are predominantly conducted in late spring or early summer, favoring early‐season species. Researchers should consider whether the phenology of species used for nondetection data complements that of focal species when using approaches similar to ours. Future iterations of RPBB modeling could compare our estimates of occupancy dynamics informed by community science data for common *Bombus* species with true nondetections of RPBB from structured monitoring data.

Since the listing of RPBB as an endangered species, no formal analysis of RPBB occupancy trends has been performed (USFWS, 2022b). Our results further demonstrate the risk of relying on raw detections when inferring changes in species distribution or tracking recovery goals (Yoccoz et al., 2001). Both the number of raw RPBB detections and counts of grid cells with detections showed a rapid increase during our study period, primarily due to increased community science efforts, but combining RPBB presence‐only data with common *Bombus* surveys revealed occupancy estimates remained static, or decreased slightly, during this period. Thus, our research provides additional insights into RPBB recovery progress that would be obfuscated by raw counts. The RPBB occupancy estimates were higher than naïve estimates, suggesting there are grid cells where the species is relatively likely to occur, but limited sampling effort has been dedicated to look for bumble bees. Our model can be easily extended to identify these grid cells for targeted survey efforts by community science groups. Identifying new RPBB locations is a priority goal in the RPBB recovery implementation strategy (USFWS, 2022a).

Our modeling framework can be used to evaluate progress toward achieving the recovery goals listed in the RPBB Recovery Plan (USFWS, 2021). The USFWS recovery plan states that each of the 5 conservation units must have “a stable or increasing trend in percent occupancy over a minimum of 5–10 years” before the species can be considered for downlisting under the ESA. Despite a nearly 150% increase in RPBB detections from 2017 to 2022, our results revealed that RPBB occupancy trends either remained static or decreased slightly across this period in Conservation Units 1 and 2 (Figure 3). A recent genetic study cautioned against inferring population stability when RPBB occurrence at a local scale is consistent across years due to low colony abundance across the RPBB range (Mola et al., 2024). Results from our study can be used to identify areas where RPBBs are infrequently or rarely detected; these areas may be important for maintaining connectivity among separate populations thereby reducing isolation (Mola et al., 2024). Progress toward recovery goals of endangered species needs to be routinely reevaluated using methods that address primary sources of variability in monitoring data (Campbell et al., 2002; Martin et al., 2007). Our approach provides a mechanism for decision makers to generate annual estimates of RPBB occupancy across all conservation units and evaluate temporal trends in RPBB occupancy in the absence of structured monitoring data. Our model can easily be extended to other conservation units with increased sampling or as new populations of RPBBs are discovered, such as the Appalachian Mountains in Conservation Unit 4 (Hepner et al., 2024).

Climate change and drought are global threats to pollinator conservation in the 21st century (Marshman et al., 2019; Vasiliev & Greenwood, 2021). Although modeling climate stressors was not the main focus of our study, our results provide preliminary evidence of a mechanism by which drought may shape the distribution of RPBBs in the Midwest, specifically by increasing local extirpation (i.e., occupied grid cells becoming unoccupied). Our study supports a growing body of research demonstrating the effects of drought on declining bumble bees (Graves et al., 2020; Janousek et al., 2023) and highlights the need for additional research into the effects of climate change on native bees. Sustained drought reduces forb productivity and thus limits nectar and pollen resources for bumble bees (Phillips et al., 2018). Colonization rates (i.e., unoccupied cells becoming occupied) did not increase during the period of deluge, potentially suggesting that RPBB populations require several years to recover from a single drought event via dispersal from other occupied areas. This finding is consistent with other research that shows bumble bee populations respond negatively to drought and can be slow to rebound when climatic conditions are more favorable (Janousek et al., 2023; Soroye et al., 2020). Colony abundances of RPBBs are low enough across their range that stochastic events (such as a 1‐year drought) could lead to extirpations (Mola et al., 2024).

Our results did show evidence of a metapopulation rescue effect (Hanski, 1998), in which grid cells surrounded by a high number of occupied neighbors are more likely to be recolonized and RPBBs are more likely to persist (i.e., occupied cell remains occupied). Thus, it appears clusters of occupied grid cells, such as those in southcentral Wisconsin (Figure 4), may be viewed as important areas for continued conservation efforts. Indeed, our results showed extirpation probabilities can be extremely high for some grid cells, highlighting the importance of having a high number of occupied neighbors to facilitate recolonization.

Our modeling framework can be extended to include other stressors, such as land‐use change, habitat fragmentation, pesticide use, and projected climate scenarios, to forecast such impacts on the distribution of RPBBs and other *Bombus* species being considered for listing under the ESA. Pathogen spread likely played a significant role in the decline of several *Bombus* species (Cameron et al., 2011). Continued development of genetic methods and eDNA technology will make it easier to obtain real‐time data on the movement of pathogens in extant *Bombus* populations (Cameron et al., 2016; Cordes et al., 2012), making it easier to incorporate pathogen threats in distribution models that account for imperfect detection of bumble bees and their pathogens (McClintock et al., 2010). Our results showed sampling efforts for bumble bees are highly concentrated in urban systems, largely due to the significant increase in community science efforts. Given that urban areas are warming more rapidly than rural areas (Rizwan et al., 2008), and these urban warming events can negatively affect pollinators (Hamblin et al., 2018), it is unclear whether urban centers will continue to serve as refugia for RPBBs in the future. Additional sampling and repeated visits in rural systems are needed before the conservation community has a clear picture of the distribution of RPBBs, and most other pollinators, and will help clarify whether drivers of occupancy dynamics, such as drought, differ between rural and urban locations. Results from our study can be combined with finer scale habitat association data (Boone et al., 2023b) and spatially explicit community science records of RPBB‐preferred plants (Wolf et al., 2022) to guide surveyors to specific locations to look for new populations of RPBBs. First identifying preferred plants may be particularly important in the eastern United States where RPBB sightings are exceptionally rare (Hepner et al., 2024).

## Supporting information



Supporting Information
