# Supplementary material for: Integrating data to assess occupancy patterns of an endangered bumble bee
Source: Conserv Biol. 2025 Feb 25;39(4):e14458. doi: 10.1111/cobi.14458 (PMC12309646; doi:10.1111/cobi.14458)

Appendix S1. Comparison of relative support among preliminary models evaluating effects of land cover covariates on initial occupancy probabilities $\psi_{1}$ of rusty-patched bumble bees *Bombus affinis* in the Upper Midwest United States. We used a simplified dynamic occupancy model with year-specific intercepts on colonization and extirpation probabilities and a time-varying structure for detection probability (detection probability varied by year and survey week). Covariates included in candidate models include proportions of grassland/herbaceous (Herbaceous), pasture/hay (Hay_Pasture), cultivated crops (Crops), shrub/scrub (Shrub_Scrub), development open space (Open_Dev), development low intensity (Low_Dev), sum of development open space and low intensity (OpenLow_Dev), sum of development open space, low intensity, and medium intensity (OpenLowMed_Dev), sum of development low intensity and medium intensity (LowMed_Dev), sum of all development subclassifications (All_Dev). WAIC = Widely Applicable Information Criterion.

| Model structure for $\psi_{1}$ | WAIC | ΔWAIC |
| --- | --- | --- |
| OpenLowMed_Dev + Herbaceous + Hay_Pasture | 3564.63 | 0 |
| OpenLowMed_Dev + Herbaceous + Hay_Pasture + Shrub_Scrub | 3566.80 | 2.16 |
| LowMed_Dev + Herbaceous + Hay_Pasture + Crops | 3567.02 | 2.38 |
| OpenLowMedDev + Hay_Pasture + Crops + Shrub_Scrub | 3567.53 | 2.90 |
| Open_Dev + Herbaceous + Hay_Pasture + Crops | 3567.54 | 2.91 |
| OpenLowMed_Dev + Herbaceous + Hay_Pasture + Crops + Shrub_Scrub | 3568.26 | 3.63 |
| Open_Dev + Herbaceous + Hay_Pasture + Crops + Shrub_Scrub | 3568.34 | 3.71 |
| LowMed_Dev + Hay_Pasture + Crops + Shrub_Scrub | 3568.56 | 3.93 |
| LowMed_Dev + Herbaceous + Hay_Pasture + Crops + Shrub_Scrub | 3568.79 | 4.15 |
| Open_Dev + Hay_Pasture + Crops + Shrub_Scrub | 3569.71 | 5.08 |
| All_Dev + Herbaceous + Hay_Pasture + Crops + Shrub_Scrub | 3570.20 | 5.57 |
| Low_Dev + Herbaceous + Hay_Pasture + Crops + Shrub_Scrub | 3570.48 | 5.84 |
| Herbaceous + Hay_Pasture | 3573.35 | 8.72 |
| LowMed_Dev + Herbaceous + Hay_Pasture | 3574.98 | 10.35 |
| OpenLowMed_Dev + Herbaceous + Hay_Pasture + Crops | 3575.93 | 11.30 |
| OpenLow_Dev + Herbaceous + Hay_Pasture + Crops + Shrub_Scrub | 3578.16 | 13.53 |
| LowMed_Dev + Herbaceous + Hay_Pasture + Shrub_Scrub | 3579.08 | 14.45 |
| Open_Dev + Herbaceous + Hay_Pasture | 3579.45 | 14.82 |
| Open_Dev + Herbaceous + Hay_Pasture + Shrub_Scrub | 3581.30 | 16.67 |
| Herbaceous + Hay_Pasture + Crops + Shrub_Scrub | 3581.79 | 17.16 |
| Herbaceous + Hay_Pasture + Shrub_Scrub | 3583.02 | 18.39 |
| Crops + Shrub_Scrub | 3591.68 | 27.05 |
| Open_Dev + Herbaceous + Crops + Shrub_Scrub | 3594.81 | 30.18 |
| LowMed_Dev + Herbaceous + Crops + Shrub_Scrub | 3595.68 | 31.05 |
| OpenLowMed_Dev + Herbaceous + Crops + Shrub_Scrub | 3595.88 | 31.25 |

Appendix S2. Palmer Drought Severity Index (PDSI) values in grid cells surveyed for rusty-patched bumble bees and other *Bombus* species across our study period (2017 – 2022) in the Upper Midwest United States. Positive values indicate wetter than average conditions and negative values indicate dryer than average conditions.


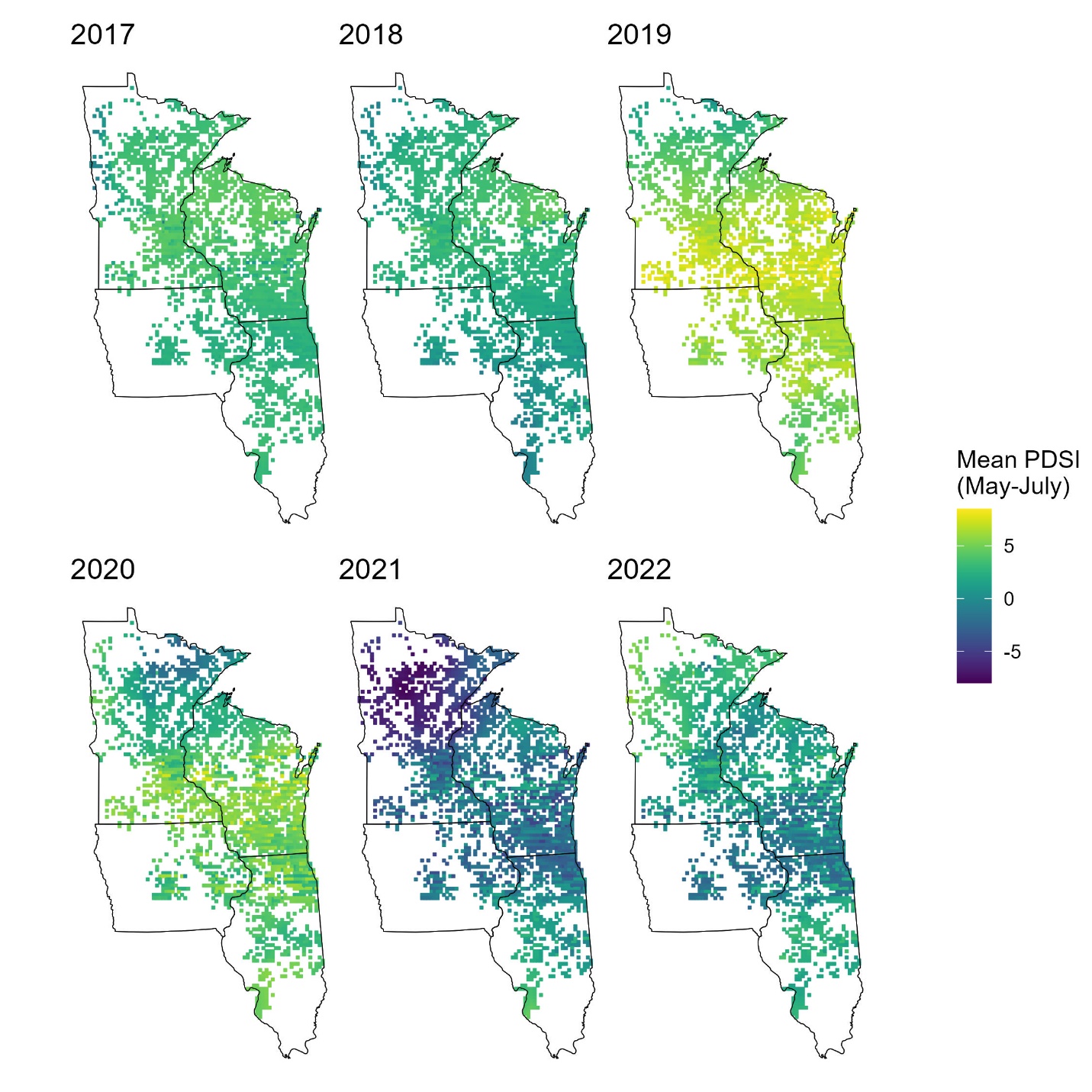


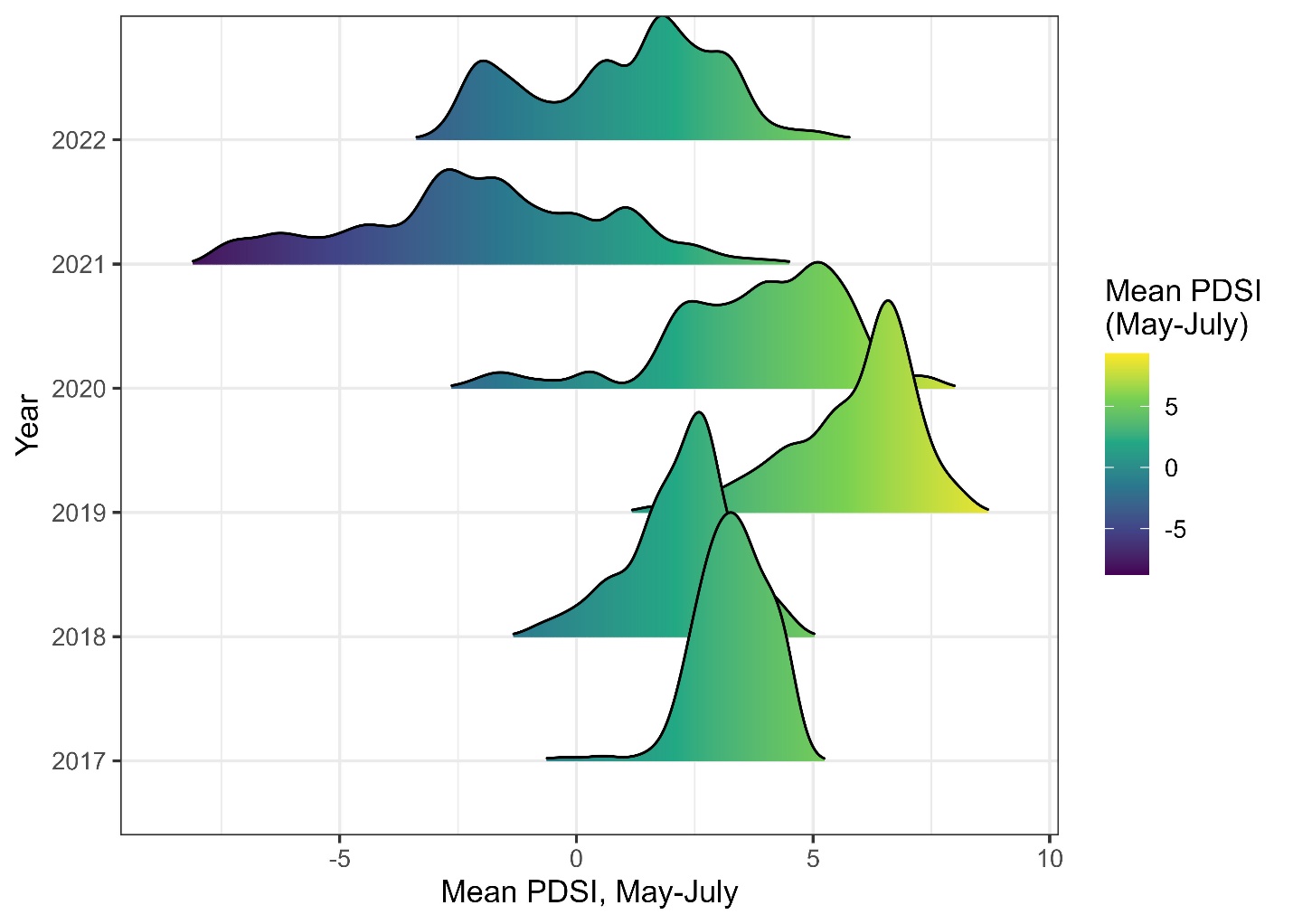


Appendix S3. Proportions of surveyed grids with rusty-patched bumble bee (RPBB) *Bombus affinis* detections (naïve occupancy), the number of grids with detections, and the number of grids surveyed for common *Bombus* spp. and rusty patched bumble bees between 2017 and 2022 in the Upper Midwest United States.

|  | Naïve occupancy (Grids with RPBB detections/Surveyed grids) | | |
| --- | --- | --- | --- |
| Year | Conservation Unit 1 | Conservation Unit 2 | All sampling grids |
| 2017 | 0.19 (62/324) | 0.19 (28/146) | 0.19 (90/470) |
| 2018 | 0.20 (92/453) | 0.36 (83/228) | 0.26 (175/681) |
| 2019 | 0.11 (67/630) | 0.13 (37/284) | 0.11 (104/914) |
| 2020 | 0.14 (100/732) | 0.14 (53/376) | 0.14 (153/1,108) |
| 2021 | 0.20 (162/807) | 0.17 (69/412) | 0.19 (231/1,219) |
| 2022 | 0.20 (182/918) | 0.10 (38/385) | 0.17 (220/1,303) |
| All years | 0.19 (321/1,665) | 0.23 (165/711) | 0.20 (486/2,376) |

Appendix S4. Posterior distributions of finite-sample occupancy probabilities (conditional on the sampled grid cells) for rusty-patched bumble bees between 2017 and 2022 in Conservation Units 1 and 2 and across the entire sampled area (Population) in the Upper Midwest United States. Points show posterior means with 66% (thick vertical bars) and 95% (thin vertical bars) credible intervals.


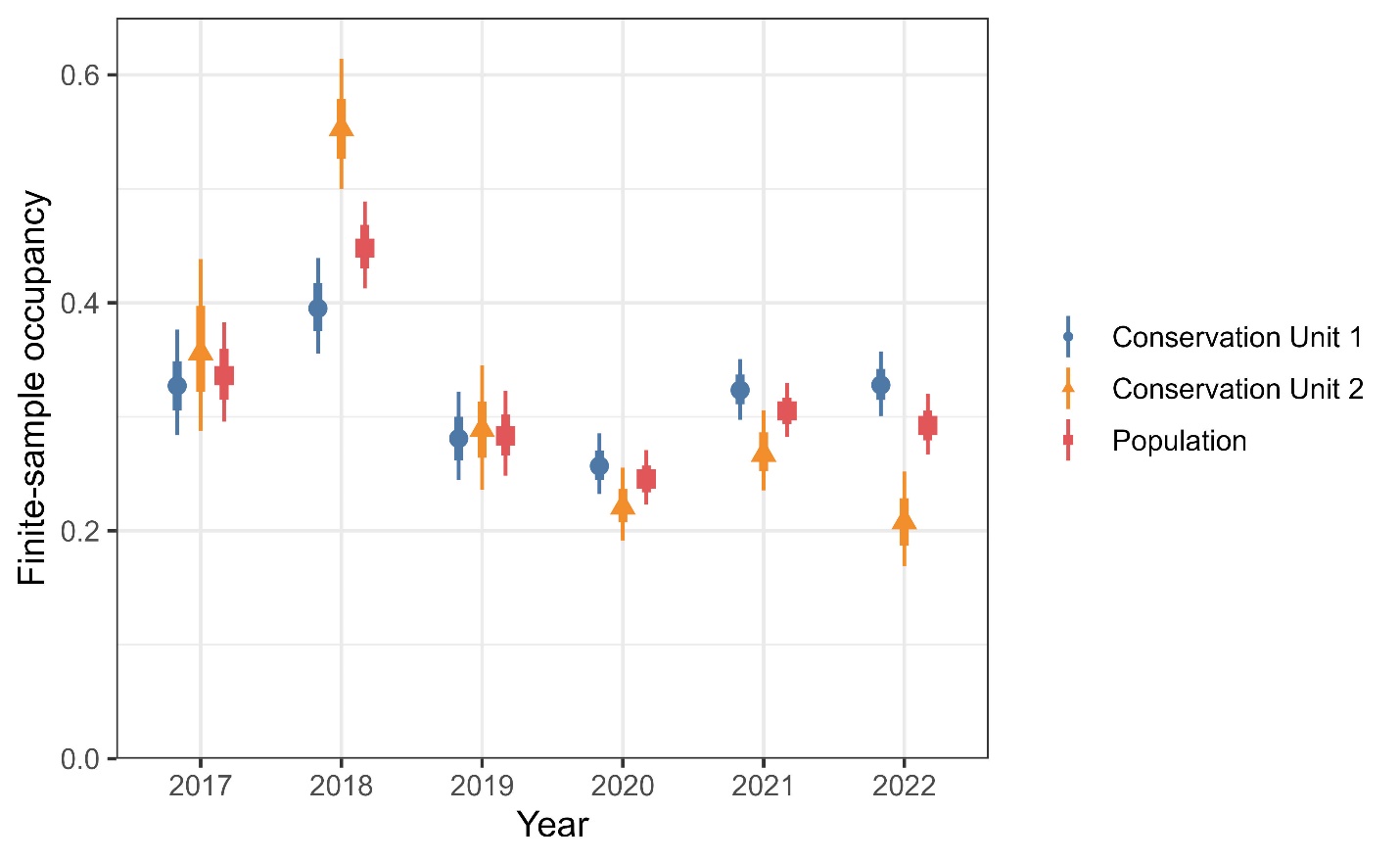


Appendix S5. Estimated number of occupied grid cells for rusty-patched bumble bees between 2017 and 2022 in Conservation Units 1 and 2 and across the entire sampled area (Population) in the Upper Midwest United States. Conservation Units 1 and 2 contain 1,665 and 711 sampled grids, respectively. Points show posterior means with 66% (thick vertical bars) and 95% (thin vertical bars) credible intervals.


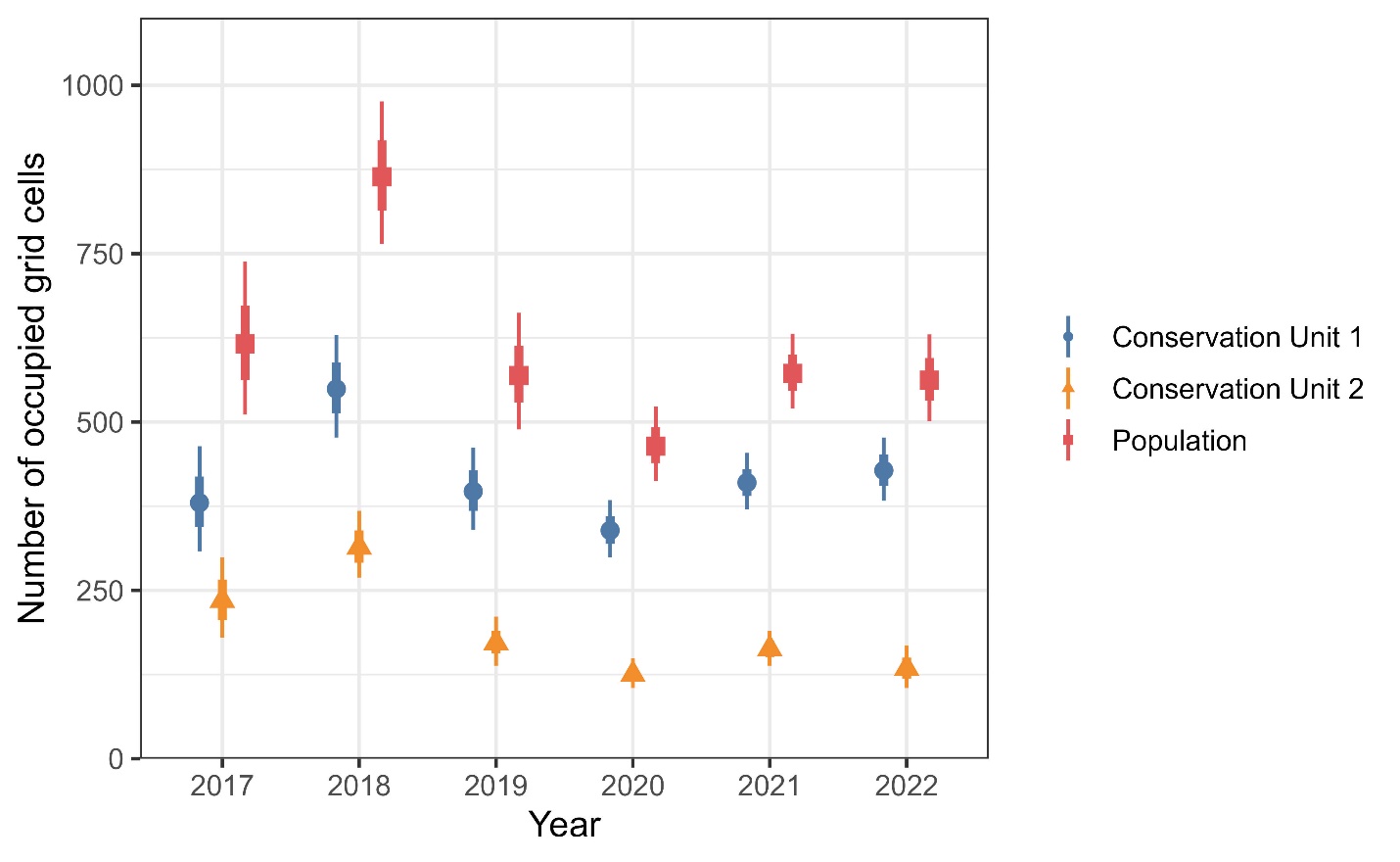


Appendix S6. Posterior means and 95% credible intervals for intercepts and covariate coefficients explaining variation in probabilities of detection, initial occupancy, colonization, and extirpation for rusty-patched bumble bees (*Bombus affinis*) between 2017 and 2022 in the Upper Midwest United States. Conservation Unit was modeled as an indicator variable where Conservation Unit 1 = 1 and Conservation Unit 2 = 0. Covariates for initial occupancy were proportions of grassland or herbaceous (herb), pasture or hay (hay), development (DEV). PDSI = Palmer Drought Severity Index.

| Parameter | Covariate | Coefficient mean | 95% CI |
| --- | --- | --- | --- |
| Detection | Count | 0.08 | (0.06, 0.09) |
| Initial occupancy | Intercept | -0.80 | (-1.31, -0.32) |
|  | Herb | 0.12 | (-0.08, 0.34) |
|  | DEV | 0.24 | (0.09, 0.39) |
|  | Hay | 0.85 | (0.63, 1.08) |
|  | Conservation Unit 1 | -0.58 | (-1.07, -0.07) |
| Colonization | Intercept - 2018 | -2.74 | (-3.66, -1.91) |
|  | Intercept - 2019 | -4.06 | (-5.38, -2.89) |
|  | Intercept - 2020 | -5.70 | (-7.31, -4.44) |
|  | Intercept - 2021 | -3.59 | (-4.54, -2.74) |
|  | Intercept - 2022 | -3.39 | (-4.21, -2.68) |
|  | Neighbors | 0.94 | (0.76, 1.14) |
|  | PDSI | -0.09 | (-0.54, 0.37) |
|  | Conservation Unit 1 | -0.34 | (-0.92, 0.23) |
| Extirpation | Intercept - 2018 | -1.44 | (-3.01, -0.31) |
|  | Intercept - 2019 | 2.04 | (1.03, 3.15) |
|  | Intercept - 2020 | 1.50 | (0.60, 2.47) |
|  | Intercept - 2021 | -1.38 | (-2.64, -0.20) |
|  | Intercept - 2022 | -0.30 | (-1.29, 0.66) |
|  | Neighbors | -0.34 | (-0.50, -0.20) |
|  | PDSI | -0.94 | (-1.59, -0.35) |
|  | Conservation Unit 1 | -0.80 | (-1.29, -0.33) |

Appendix S7. Comparison of the estimated number of occupied neighbors ($\hat{\theta}$) in each year and the observed detections in neighboring grids (top panel) for rusty-patched bumble bees in the Upper Midwest United States. The observed detections in neighboring grids were summed across all years whereas the estimated number of occupied neighboring grids was estimated separately for each year. Points show posterior means with 66% (thick vertical bars) and 95% (thin vertical bars) credible intervals. The bottom panel shows the distribution of $\hat{\theta}$ which was used as a covariate explaining variation in extirpation and colonization probabilities.


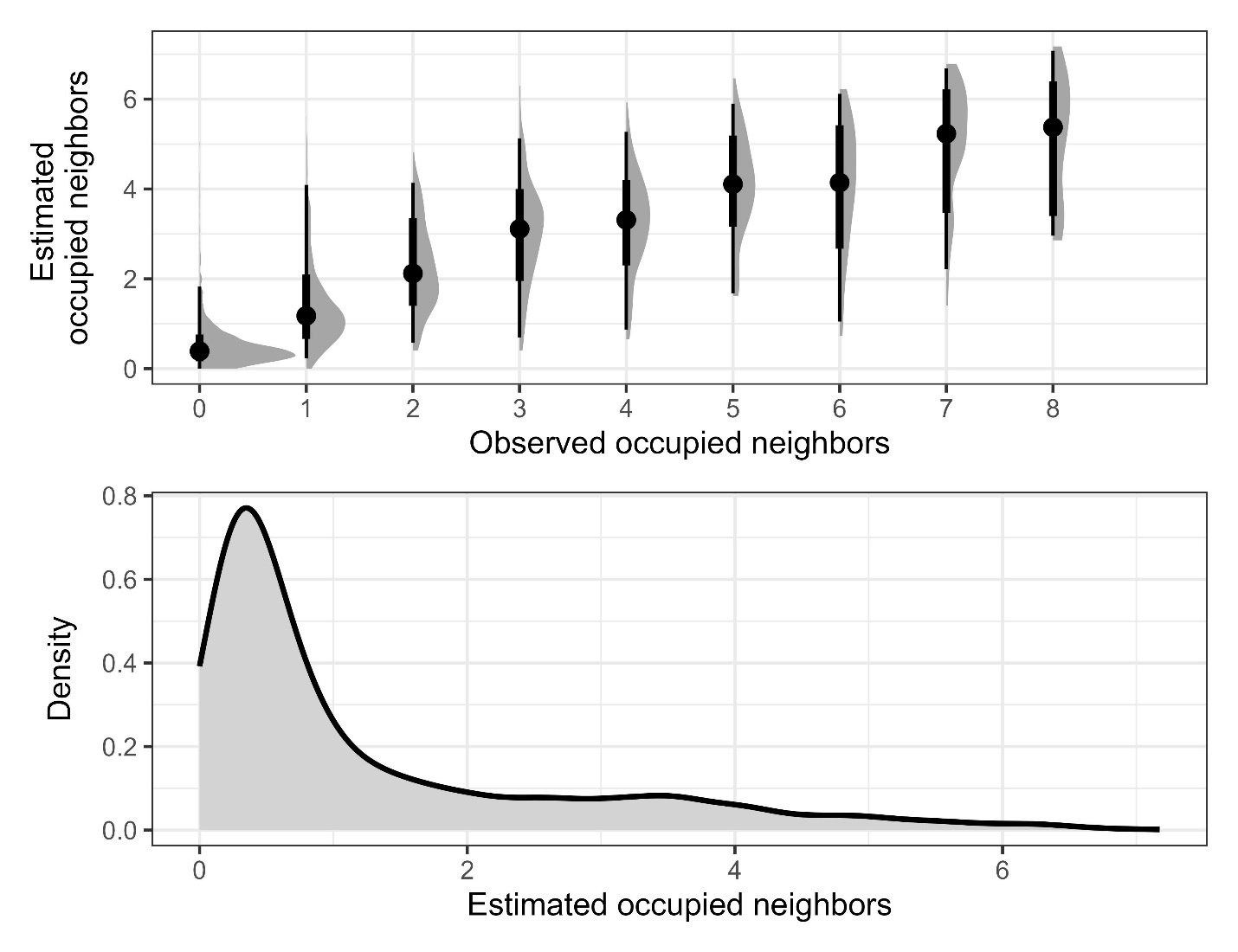


Appendix S8. Annual extirpation and colonization probabilities for rusty patched bumble bees between 2018 and 2022 in Conservation Units 1 and 2 (denoted by panel labels). Probabilities were calculated using the average Palmer Drought Severity Index across our study period (unstandardized PDSI = 2.17) and the average number of occupied neighbor grids ($\hat{\theta}$ = 1.42). Points show posterior means with 66% (thick vertical bars) and 95% (thin vertical bars) credible intervals.
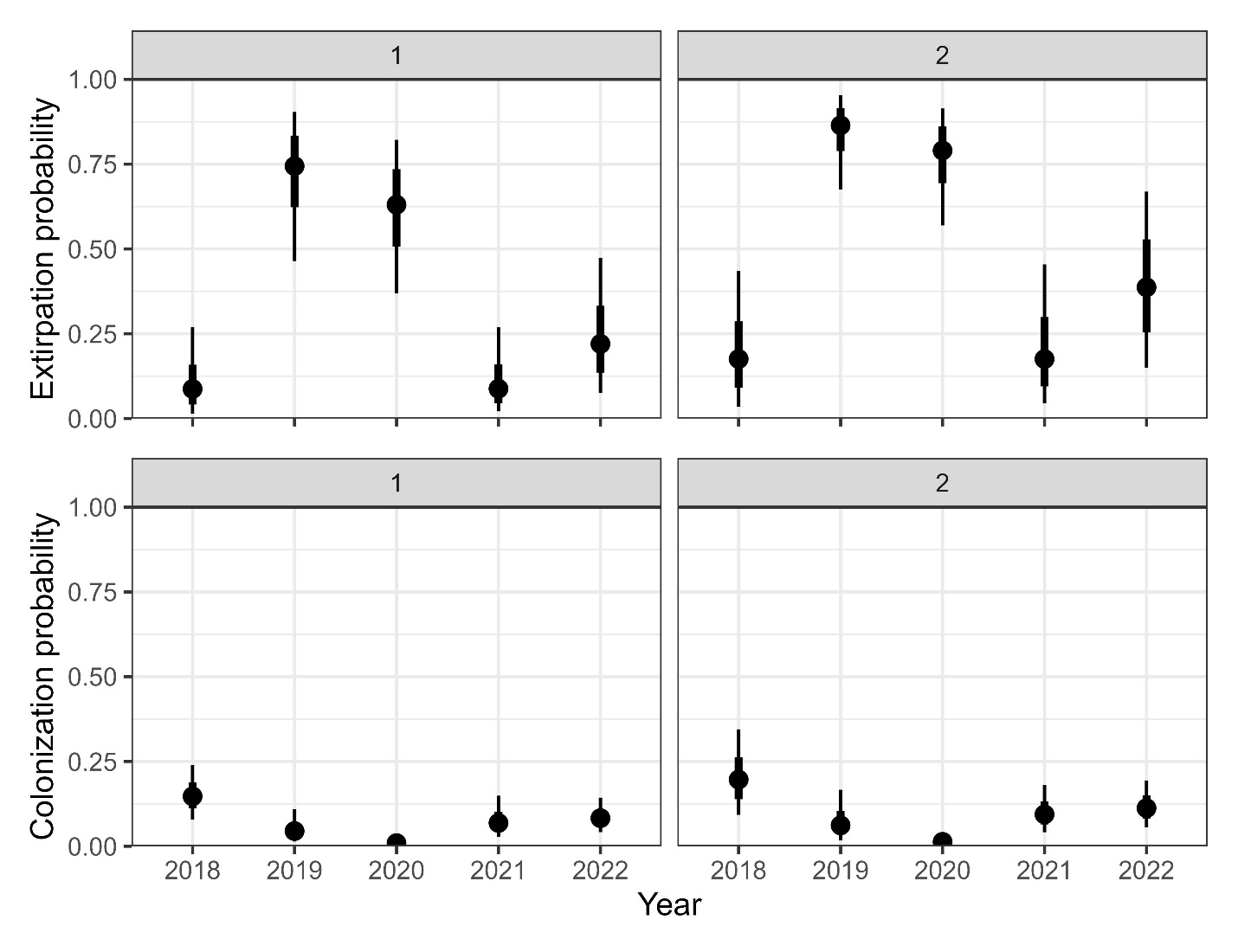


Appendix S9. Detection probabilities of rusty patched bumble bees by week between 2017 and 2022 (top). Lines show posterior mean probabilities and shading shows 95% credible intervals. The weekly standard deviation of detection probabilities ($\sigma_{p}$) equaled 0.65 (95% CI = 0.53 – 0.78) on the logit scale. The bottom panel shows posterior distributions of detection probabilities averaged across weeks in each year. Points show posterior means with 66% (thick vertical bars) and 95% (thin vertical bars) credible intervals.


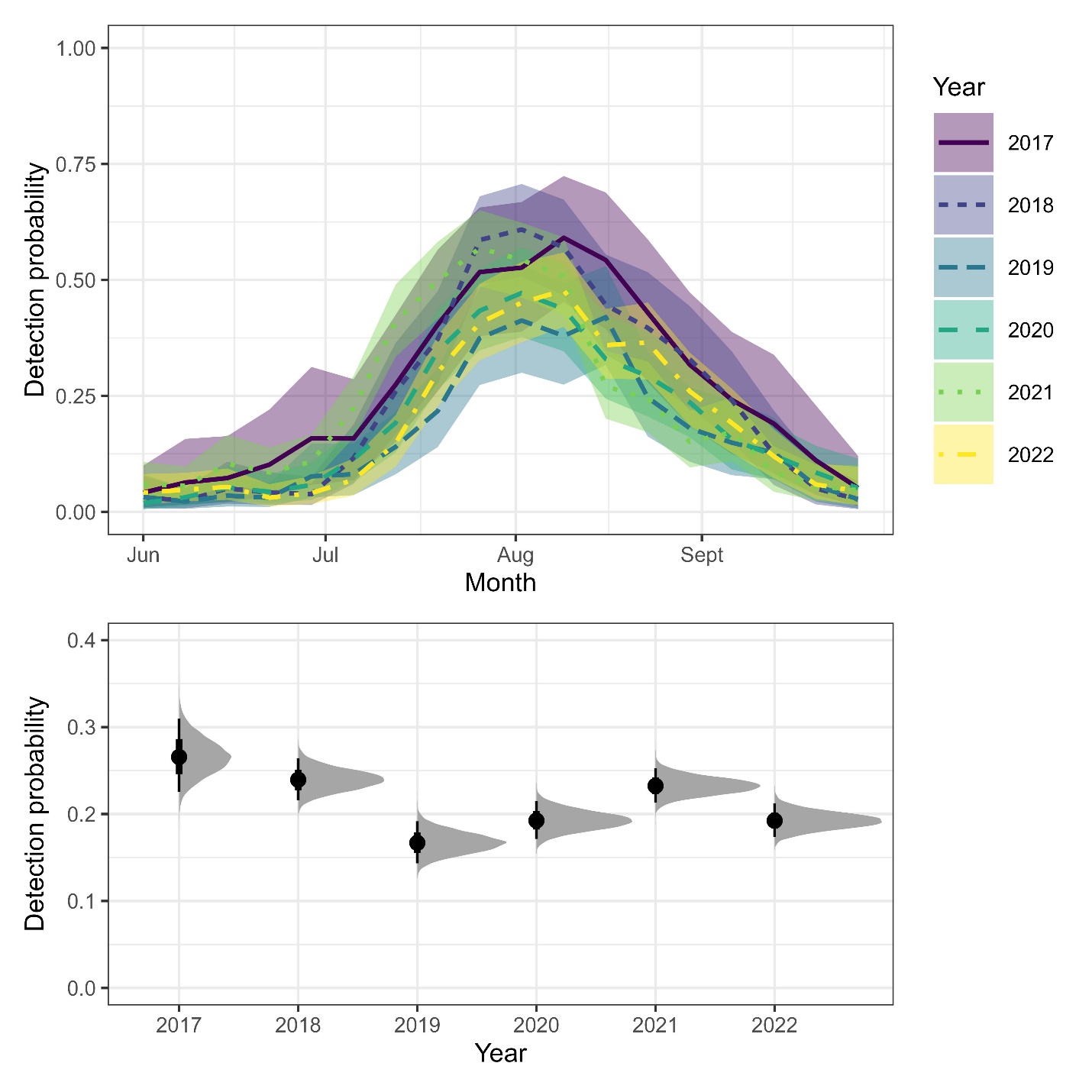

Supplement: Supplementary file 1 — Supporting Information [file COBI-39-e14458-s001.docx]
